# The War Wound and Appendicitis, an Analogy

**Published:** 1918-09

**Authors:** 


### WAR MEDICINE

Published by the American Red Cross Society in France
for the
Medical Officers of the American Expeditionary Forces

**EDITORIAL OFFICES : 9, rue du Mont-Thabor, Paris (Ier)**

WAR MEDICINE *accepts no original articles. Its scope is limited to the publication of the proceedings of the Research Society of the American Red Cross in France, to abstracts of original articles, and to editorial comment on subjects pertaining to the medicine, surgery, and hygiene of the war.*

*Circulars, bulletins, and reports from the Office of the Chief Surgeon of the American Expeditionary Forces will also appear in* WAR MEDICINE.

WAR MEDICINE *is distributed free of charge to the medical officers of the American Expeditionary Forces.*

*All communications pertaining to the mailing list should be addressed to the Editorial Offices. If copies are not received promptly and regularly the managing editor should be informed at once. Addresses should be written distinctly.*

THE RESEARCH SOCIETY OF THE AMERICAN RED CROSS IN FRANCE
*All communications should be addressed to the Secretary,*
*9, rue du Mont-Thabor, Paris (Ier)*

### EDITORIAL COMMENTS

### THE WAR WOUND AND APPENDICITIS
### AN ANALOGY

In *acute appendicitis*, if the dead appendix is removed before the infection invades normal tissue, then the wound is immediately closed. Primary union follows and the patient is detained only to heal, and the vast possibilities of the inflammation of a great cavity are averted. If the dead tissue in the track of a *war wound* is excised, before living tissue is invaded, inflammation of a great mass of soft tissue is equally averted.

In *appendicitis*, even after the infection has penetrated the wall of the appendix and invaded the peritoneum, excision of the dead appendix facilitates the recovery; in *war wounds*, even after the infection passes beyond the devitalized tissue, excision of the devitalized tissue facilitates recovery. In appendicitis, as in war wounds, if operation is performed during the stage of spreading infection, there should not, as a rule, be complete and immediate closure of the wound.

When an appendicitis has gone on to a localized abscess with an *organized* wall of granulations, then the surgeon would deal with the abscess as such; when in the war wound, the infection has gone on to new tissue organization and abscess, no débridement is done, but the abscess is treated as such. In the war wound, we find that the stage of contamination — i.e., before infection — of dead tissue corresponds to the period of time before the infection of a dead appendix penetrates the wall. Excise the dead appendix cleanly and the case recovers; excise the dead tissue in the war wound cleanly, and it recovers. In each, the process goes through identical stages — localized infection, spreading infection, abscess.

It was only after the surgeon learned to forecast accurately the *tomorrow* of the acute appendicitis and acted *today*; only after he took the initiative away from the disease, and attacked instead of defended, that he mastered appendicitis. As long as the surgeon waits to see what the war wound means to do, leaving the initiative to the infection, just so long will he fail to achieve mastery. To wait in the case of a devitalized war wound until the infection is spreading wildly — until there is pain, swelling, redness and tenderness — is to come to the rescue of the living tissue after it has been partially defeated. We must read the tomorrow of each wound accurately — read it and act on that reading now. We do not offer appendicitis an antiseptic — we offer it surgery.

Evidence is accumulating that the fever and pain and tenderness and swelling can be cut out of an infected wound under battle conditions. We have now seen that the mortality and the morbidity may within certain limits be reclaimed up to the third day, just as in appendicitis. The fourth day in the war wound, as in appendicitis, is the critical day. Why are operations so successful up to the fourth day? And why in the face of intense infection is the wound repair so rapid? Why is it possible successfully to close acute abdomens with much infection? Why do the neglected, contaminated, but excised war wounds heal so well when the slightest infection causes failure in an operation for hernia? Because, in the case of the war wound, the local defenses have been called out actively; in the case of the hernia they have not. In the revised war wound there is increased blood supply, increased serum and increased phagocytosis. It is safer to have a breach in the aseptic technique in operations in an area whose defense is already heightened than in an area whose defense has not been heightened by injury.

The analogy does not end here, for if the appendix abscess is roughly handled there is caused spreading peritonitis, pylophlebitis, bacteriema, etc. If the infected war wound, especially if it has gone to abscess, is roughly handled, there is apt to be spreading infection, bacteriema, etc.

A war wound is appendicitis of the thigh, the leg, the arm. There is no difference in principle or in practice between the treatment of appendicitis and the treatment of war wounds. The time factor holds; the treatment is comparable; the natural defense identical.

